# Reevaluating the neuroprotective promise of dietary nitrate: Commentary on Rajendra et al. (2025)

**DOI:** 10.1016/j.tjpad.2025.100339

**Published:** 2025-08-25

**Authors:** Parth Aphale, Himanshu Shekhar, Shashank Dokania

**Affiliations:** Dr. D.Y. Patil Vidyapeeth, Deemed to be University, Pimpri, Pune, Maharashtra, India

To the Editor,

We read with great interest the article by Rajendra et al., *“Baseline habitual dietary nitrate intake and Alzheimer’s Disease related neuroimaging biomarkers in the AIBL study”* [1]. The authors are to be commended for their rigorous longitudinal analysis over 126 months, integrating dietary nitrate intake with advanced neuroimaging outcomes in a well-characterized cohort. Their stratified approach, particularly by APOE ε4 genotype and sex, reflects a nuanced understanding of Alzheimer’s disease (AD) heterogeneity and is a welcome advancement in nutritional epidemiology. The finding that higher plant-sourced nitrate intake is associated with attenuated cerebral β-amyloid (Aβ) deposition and hippocampal atrophy—especially among female APOE ε4 carriers—is both novel and potentially impactful. This sex- and genotype-contingent insight enriches our understanding of dietary modulation in AD prevention and contributes meaningfully to the growing field of precision nutrition.

Nonetheless, several **methodological and interpretive considerations** merit discussion to contextualize these findings.

First, although the authors acknowledge variability in nitrate bioavailability and potential adverse effects of nitrate-nitrite conversion, the study does not sufficiently address the **biochemical plausibility** underlying the differential impact of plant- versus animal-sourced nitrate. The inference of plant nitrate’s neuroprotective potential—while biologically conceivable via nitric oxide (NO)-mediated vasodilation and perfusion—is not directly validated in this cohort through intermediary biomarkers such as serum NO metabolites or cerebrovascular function indices [2,3].

Second, while the **use of Centiloid-scaled PET imaging** and harmonized MRI protocols ensures standardization, the reliance on **tertile-based nitrate exposure classification** risks obscuring nonlinear dose–response relationships. The observation of protective effects primarily in the “moderate” intake groups (rather than highest tertiles) suggests potential **U-shaped associations**, which warrant more robust modeling using continuous exposure variables or restricted cubic splines [4].

Third, the adjustment models, though extensive, may not fully mitigate **residual confounding** from overall dietary patterns or unmeasured lifestyle variables. Despite including dietary fat, red meat, and alcohol intake as covariates, the complex interplay between nitrate-rich vegetable consumption and broader cardiometabolic dietary profiles may introduce bias—especially in observational settings [5]. Moreover, the **Food Frequency Questionnaire (FFQ)**, although validated, remains vulnerable to recall bias and misclassification, especially in elderly populations .

Lastly, while the study prudently stratified by **APOE ε4 genotype**, it did not explore **potential interactions between nitrate intake and vascular comorbidities** (e.g., hypertension, diabetes), which could further mediate or confound the observed neuroimaging associations .

In conclusion, the authors present a valuable contribution to the literature on dietary nitrate and AD risk, particularly through the lens of APOE-specific and sex-stratified neuroimaging outcomes. However, the causal interpretation of these associations should remain cautious in the absence of biomarker triangulation or randomized dietary interventions. Future studies incorporating cerebrovascular measures, nitrate metabolism indicators, and cognitive endpoints are needed to confirm these intriguing findings.

## CRediT authorship contribution statement

**Parth Aphale:** Conceptualization, Supervision, Writing – original draft, Writing – review & editing. **Himanshu Shekhar:** Conceptualization, Writing – original draft, Writing – review & editing. **Shashank Dokania:** Writing – original draft, Writing – review & editing.

## Declaration of competing interest

The authors declare that they have no known competing financial interests or personal relationships that could have appeared to influence the work reported in this paper.
